# Supplementary material for: A Critical View of the Application of the APEX Software (Aqueous Photochemistry of Environmentally-Occurring Xenobiotics) to Predict Photoreaction Kinetics in Surface Freshwaters
Source: Molecules. 2019 Dec 18;25(1):9. doi: 10.3390/molecules25010009 (PMC7017383; doi:10.3390/molecules25010009)
Supplement: Supplementary file 1 [file molecules-25-00009-s001.zip › 3.2.4_gcc-4.4.0.zip!/3.2.4_gcc-4.4.0/3.2.4_gcc-4.4.0/doc/octave/html/interpreter/Managing-Default-Properties.html]

Managing Default Properties - Untitled


Next: Colors,
Previous: Graphics Object Properties,
Up: Advanced Plotting


---

#### 15.2.3 Managing Default Properties

Object properties have two classes of default values, factory
defaults (the initial values) and user-defined defaults, which
may override the factory defaults.

Although default values may be set for any object, they are set in
parent objects and apply to child objects. For example,

```
     set (0, "defaultlinecolor", "green");
```

sets the default line color for all objects. The rule for constructing
the property name to set a default value is

```
     default + object-type + property-name
```

This rule can lead to some strange looking names, for example
`defaultlinelinewidth"` specifies the default `linewidth`
property for `line` objects.

The example above used the root figure object, 0, so the default
property value will apply to all line objects. However, default values
are hierarchical, so defaults set in a figure objects override those
set in the root figure object. Likewise, defaults set in axes objects
override those set in figure or root figure objects. For example,

```
     subplot (2, 1, 1);
     set (0, "defaultlinecolor", "red");
     set (1, "defaultlinecolor", "green");
     set (gca (), "defaultlinecolor", "blue");
     line (1:10, rand (1, 10));
     subplot (2, 1, 2);
     line (1:10, rand (1, 10));
     figure (2)
     line (1:10, rand (1, 10));
```

produces two figures. The line in first subplot window of the first
figure is blue because it inherits its color from its parent axes
object. The line in the second subplot window of the first figure is
green because it inherits its color from its parent figure object. The
line in the second figure window is red because it inherits its color
from the global root figure parent object.

To remove a user-defined default setting, set the default property to
the value `"remove"`. For example,

```
     set (gca (), "defaultlinecolor", "remove");
```

removes the user-defined default line color setting from the current axes
object.

Getting the `"default"` property of an object returns a list of
user-defined defaults set for the object. For example,

```
     get (gca (), "default");
```

returns a list of user-defined default values for the current axes
object.

Factory default values are stored in the root figure object. The
command

```
     get (0, "factory");
```

returns a list of factory defaults.
